# Hypofibrinogenemia is an independent predictor of hemophagocytic lymphohistiocytosis in children with sepsis

**DOI:** 10.1038/s41598-023-44628-z

**Published:** 2023-10-20

**Authors:** Xin Luo, Chentao Zhou, Cunwei Ji, Chunmin Lu, Yasha Luo, Zhenhui Chen, Tianhua Zhong, Ruoting Ye, Liwei Zeng, Mingyong Luo

**Affiliations:** 1 Department of Laboratory Medicine, Guangdong Women and Children Hospital, No. 453 Xing-nan Avenue, Guangzhou, 511400 People’s Republic of China; 2 Laboratory Medicine, Medical College of Jiaying University, Guangzhou, 511400 People’s Republic of China

**Keywords:** Diseases, Risk factors, Signs and symptoms

## Abstract

Hemophagocytic lymphohistiocytosis (HLH) is a potentially life-threatening condition in children with sepsis. We herein aimed to identify clinical and laboratory predictors of HLH in children with sepsis. We conducted a retrospective study of 568 children with sepsis admitted to Guangdong Women and Children Hospital from January 2019 to June 2022. HLH, while rare (6.34%), proved to be a highly fatal complication (37.14%) in children with sepsis. Children with HLH had higher levels of aspartate aminotransferase, lactate dehydrogenase, triglycerides, and ferritin than children without HLH; conversely, they displayed decreased levels of neutrophils, hemoglobin, platelets, fibrinogen, and albumin. Additionally, the HLH group showed higher rates of prolonged fever (> 10 days), hepatomegaly, and splenomegaly than the non-HLH group. Our retrospective analysis identified hypofibrinogenemia (OR = 0.440, *P* = 0.024) as an independent predictor for the development of HLH in patients with sepsis. The optimal cutoff value for fibrinogen was found to be < 2.43 g/L. The area under the curve for diagnosing HLH was 0.80 (95% confidence interval: 0.73–0.87,* P* < 0.0001), with a sensitivity of 72.41% and specificity of 76.27%. Thus, hypofibrinogenemia emerges as a potentially valuable predictor for HLH in children with sepsis.

## Introduction

Hemophagocytic lymphohistiocytosis (HLH) is a life-threatening syndrome, often caused by infectious agents. It is characterized by an uncontrolled and ineffective immune response, resulting in a state of immune hyperactivation. Key clinical manifestations include fever, splenomegaly, and cytopenia. Primary HLH is recognized as an inherited genetic disorder, primarily stemming from mutations in genes related to cytotoxic granule activity pathways^1^. Secondary HLH emerges in the context of infections, malignancies, and autoimmune conditions. Owing to its nonspecific symptoms and resemblance to other inflammatory disorders, HLH diagnosis is frequently delayed and prone to misdiagnosis.

Sepsis stands as a predominant cause of mortality in pediatric intensive care units. Sepsis is defined as a life-threatening condition marked by organ dysfunction resulting from a dysregulated host response to infection^2^. Both primary and secondary HLH can be triggered by various infections, including viruses, bacteria, and fungi^3,4^. Clinical and laboratory features shared by HLH and sepsis include fever, hyperferritinemia, hemophagocytosis in bone marrow, and multiorgan dysfunction. However, the treatment approaches for sepsis and HLH differ significantly. Sepsis management primarily involves anti-infectious agents and intensive symptomatic therapy. In contrast, primary HLH necessitates bone marrow transplantation, and secondary HLH is managed through chemotherapy and addressing the underlying disease. Consequently, identifying clinical or laboratory predictors that facilitate early HLH diagnosis in children with sepsis is of paramount importance.

In the present study, we conducted a comprehensive review of medical records of patients diagnosed with sepsis. The objective of this investigation was to investigate potential predictors of HLH within patients with sepsis.

## Results

### Patient characteristics

A total of 568 children with sepsis (231 girls and 337 boys; female-to-male ratio: 0.69) were included in this study between January 2019 and June 2022. Their ages ranged from 0 to 14 years, with an average age of 1.92 years. Among these patients, 36 were diagnosed with HLH during their hospitalizations, yielding an HLH incidence of 6.34%. The median age in the HLH group was 1 year, which was not significantly different from patients without HLH (n = 532, 1.5 years, *P* = 0.4041). Of the 36 patients with HLH, 23 were males and 13 were females. The proportion of males among children with HLH did not significantly differ from that of patients without HLH (53.92% vs. 63.89%, *P* = 0.6042).

### Clinical characteristics between patients with sepsis and HLH

Table 1 presents the clinical characteristics of children with and without HLH. Children with HLH exhibited significantly higher rates of hepatomegaly (30.56% vs. 5.08%, *P* < 0.0001), prolonged fever lasting more than 10 days (25% vs. 7.33%, *P* = 0.0017), and splenomegaly (16.67% vs. 1.88%, *P* = 0.0002) than those without HLH. No significant differences were observed in terms of gender, fever, lymphadenopathy, jaundice, skin rash, and septic shock between the two groups (*P* > 0.05). The short-term (30-day) mortality rate for HLH was significantly higher than that for patients without HLH (37.14% vs. 14.88%, *P* = 0.0005).

**Table 1:** Frequency of demographic and clinical features among HLH and non-HLH groups in 568 children with sepsis.

Characteristics	Non-HLH group (n = 532)	HLH group (n = 36)	*P* value
Demographic data			
Age (year), median (IQR)	1.0 (0, 3)	1.5 (0, 3)	0.4041
Male, n (%)	314 (59.02)	23 (63.89)	0.6042
Clinical manifestations			
Fever, n (%)	501 (94.17)	35 (97.22)	0.7152
Fever for > 10 days, n (%)	39 (7.33)	9 (25.00)	0.0017
Lymphadenopathy, n (%)	70 (13.16)	9 (25.00)	0.0757
Hepatomegaly, n (%)	27 (5.08)	11 (30.56)	< 0.0001
Splenomegaly, n (%)	10 (1.88)	6 (16.67)	0.0002
Jaundice, n (%)	11 (2.07)	3 (8.33)	0.0525
Skin rash, n (%)	83 (15.60)	7 (19.44)	0.4866
Septic shock	42 (7.89)	5 (13.89)	0.2075
Short-term mortality rate	79 (14.88)	13 (37.14)	0.0005
Pathogens			
Bacteria	128 (24.06)	12 (33.33)	0.2311
Virus	26 (4.89)	8 (22.22)	< 0.0001
Fungi	50 (9.39)	5 (13.89)	0.3787

Of the children with sepsis, 35.74% (203/568) were found to have one or more pathogens identified, resulting in 140 strains of bacteria, 34 strains of viruses, and 55 strains of fungi. There were no differences in bacterial/fungal infection between the HLH and non-HLH groups. However, the rate of virus-positive cases in the HLH group was higher than that in the non-HLH group (22.22% vs. 4.89%, *P* < 0.0001). All virus-positive cases in the HLH group were associated with Epstein-Barr virus. Among 26 virus-positive cases in the non-HLH group, Epstein-Barr virus was detected in 21 cases, cytomegalovirus in 4 cases and norovirus in 1 case.

### Laboratory data between patients with sepsis and HLH

Table 2 outlines the differences in laboratory test results between children with and without HLH. Children with HLH had significantly higher levels of aspartate aminotransferase (117 vs. 41 U/L, *P* < 0.0001), lactate dehydrogenase (1113 vs. 555 U/L, *P* = 0.0002), triglycerides (2.22 vs. 1.59 mmol/L,* P* = 0.0059), and ferritin (8046 vs. 339.1 ng/mL, *P* < 0.0001) than those without HLH. Conversely, children with HLH exhibited significantly lower levels of neutrophils (2.52 vs. 8.45 × 10^9^/L,* P* < 0.0001), hemoglobin (92 vs. 106 g/L, *P* = 0.0003), platelets (153 vs. 305 × 10^9^/L, *P* = 0.0015), fibrinogen (2.165 vs. 4.2 g/L, *P* = 0.0009), and albumin (29.4 vs. 33.85 g/L, *P* = 0.0136) than those without HLH.

**Table 2:** Comparison of the laboratory results among HLH and non-HLH groups.

Numerical variable	Non-HLH group	HLH group	*P* value
	M (P25, P75)	M (P25, P75)	
IL-6	58.32 (29.74, 130.10)	116.5 (47.42, 431.7)	0.2105
CRP (mg/L)	65.45 (7.23, 114.50)	27.07 (7.58, 103.27)	0.4074
PCT (ng/mL)	1.99 (0.61, 5.81)	3.685 (1.028, 8.415)	0.1155
ESR (mm/h)	47 (16.50, 77.50)	28 (4, 56)	0.0727
Neutrophils (× 10^9^/L)	8.45 (3.76, 12.59)	2.52 (0.63, 7.048)	< 0.0001
Hemoglobin (g/L)	106 (94, 117)	92 (82, 104)	0.0003
Platelets (× 10^9^/L)	305 (210, 393)	153 (44, 356)	0.0015
Fibrinogen (g/L)	4.2 (2.68, 5.82)	2.165 (1.478, 3.738)	0.0009
AST (U/L)	41 (27, 68.5)	117 (42.75, 338.5)	< 0.0001
ALT (U/L)	41 (25,70.5)	62 (26, 164)	0.1156
GGT (U/L)	46 (17, 117)	49.5 (16, 175)	0.5306
LDH (U/L)	555 (297.5, 802)	1113 (446, 3035)	0.0002
Total protein (g/L)	58.5 (53.9, 64.98)	55.6 (48.25, 63.7)	0.2008
Albumin (g/L)	33.85 (29, 68)	29.4 (25.6, 36.55)	0.0136
Triglycerides (mmol/L)	1.59 (1.11, 2.3)	2.22 (1.625, 4.608)	0.0059
Ferritin (ng/mL)	339.1 (193.4, 591.3)	8046 (2457, 15,531)	< 0.0001

IL-6, interleukin-6; CRP, C-reactive protein; PCT, procalcitonin; ESR, erythrocyte sedimentation rate; AST, aspartate aminotransferase; ALT, alanine aminotransferase; GGT, glutamyl transpeptidase; LDH, lactate dehydrogenase.

### Predictor for HLH complication in children with sepsis

To identify potential predictors of HLH, multivariate binary logistic regression analysis was performed (Table 3). HLH was used as the dependent variable, and independent variables included factors with a *P-*value of < 0.05. Univariate analysis revealed seven factors associated with HLH at the *P* < 0.05 level, including fever lasting more than 10 days, hepatomegaly, neutrophil count, hemoglobin level, fibrinogen level, albumin level, and triglyceride level. Multivariate logistic regression analysis demonstrated that fibrinogen (odds ratio = 0.440, 95% confidence interval: 0.216–0.895, *P* = 0.024) was an independent predictor of HLH in children with sepsis.

**Table 3:** Multivariate binary logistic regression analysis of clinical and laboratory predictors using hemophagocytic lymphohistiocytosis (HLH) as a dependent variable.

Variable	B	S.E.	Wald	*P* value	odds ratio	95.0% CI
Fever for > 10 days	0.978	0.908	1.160	0.281	2.660	0.448–15.780
Hepatomegaly	0.631	1.143	0.305	0.581	1.879	0.200–17.653
Neutrophils	−0.110	0.069	2.570	0.109	0.896	0.783–1.025
Hemoglobin	−0.024	0.020	1.392	0.238	0.977	0.939–1.016
Fibrinogen	−0.822	0.363	5.127	0.024	0.440	0.216–0.895
Albumin	−0.018	0.073	0.064	0.801	0.982	0.852–1.132
Triglycerides	0.539	0.276	3.816	0.051	1.715	0.998–2.946
Constant	2.969	3.018	0.968	0.325	19.469	

### Availability of fibrinogen as a diagnostic criterion for HLH in children with sepsis

**Figure 1:**
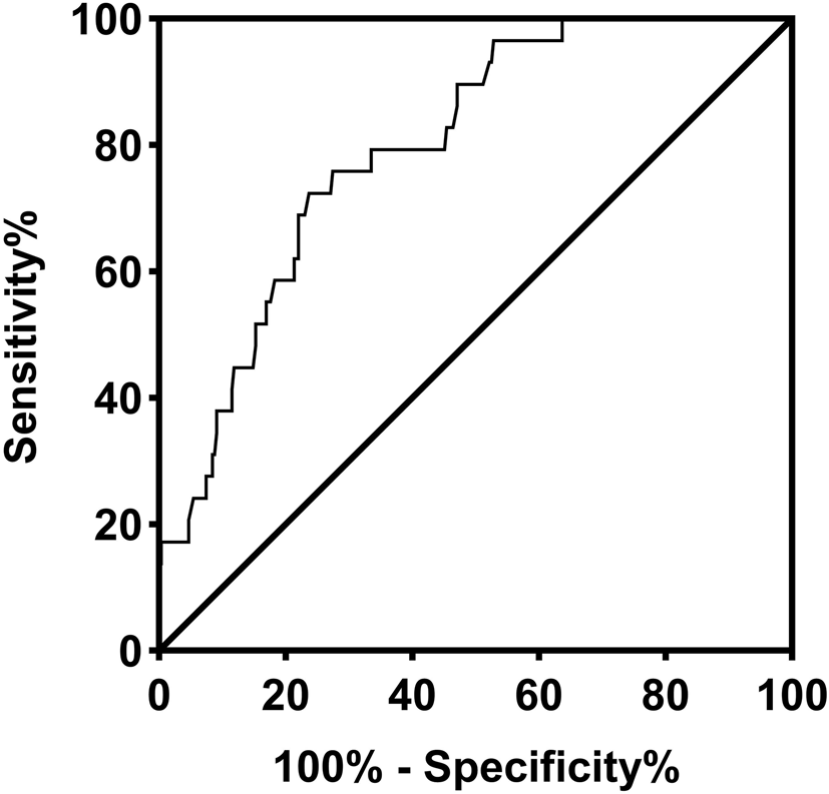
Receiver operator characteristic (ROC) curve of the serum levels of fibrinogen. The area under the curve (AUC) was 0.80 with SE = 0.04 and a 95% confidence interval (CI) from 0.73 to 0.87.

Receiver operating characteristic curve analysis was used to assess the accuracy of fibrinogen as a diagnostic criterion for HLH in children with sepsis (Fig. 1). The analysis revealed that the optimal cutoff value for fibrinogen was 2.43 g/L, resulting in an area under the curve of 0.80 (95% confidence interval: 0.73–0.87), with a sensitivity of 72.41% and a specificity of 76.27%. Reducing the cutoff value of fibrinogen to 1.5 g/L, in accordance with the diagnostic guideline for HLH, yielded a sensitivity of 31.03% and a specificity of 91.53%.

## Discussion

Patients diagnosed with HLH often represent some of the most critically ill individuals in the intensive care unit^5–7^. Given that both HLH and sepsis are syndromes characterized by potentially overwhelming inflammation, distinguishing between them poses a considerable challenge. Sepsis, defined as an infection accompanied by severe systemic inflammatory response syndrome, is a common precursor to secondary HLH in children^8–10^. However, there has been limited exploration of HLH in children with sepsis. In the present study, we uncovered an HLH incidence rate of 6.34% among pediatric sepsis cases. Importantly, the short-term mortality rate for children with HLH significantly exceeded that of children without HLH (37.14% vs. 14.88%, *P* = 0.0005), underscoring the critical need to identify predictors of HLH in pediatric sepsis.

Our study presented a substantial cohort of children with both sepsis and HLH, a group not extensively documented in the literature. Our findings revealed several distinguishing clinical and laboratory characteristics between children with sepsis with and without HLH. Notably, children with HLH exhibited significantly higher rates of prolonged fever exceeding 10 days (25% vs. 7.33%, *P* = 0.0017), hepatomegaly (30.56% vs. 5.08%, *P* < 0.0001), and splenomegaly (16.67% vs. 1.88%, *P* = 0.0002) than those without HLH. Past studies by Hanane Zahir and Feig JA reported that approximately 90% of patients with HLH presented with splenomegaly and approximately 61% with hepatomegaly^11,12^. This suggests that the presence of hepatosplenomegaly, a common feature in HLH but not sepsis, can aid in distinguishing HLH among patients with sepsis.

Fever is a hallmark symptom in HLH, reported in 90%–100% of patients and often refractory to antibiotics^13–17^. In contrast, one of the diagnostic criteria for sepsis includes hyperthermia or hypothermia, with hyperthermia being more prevalent (90%–100% of patients)^18,19^. Our study revealed that fever was the most common clinical feature in both HLH (97.22%) and non-HLH (94.17%) groups. However, the HLH group had a significantly higher rate of prolonged fever lasting over 10 days. This highlights the challenge of using fever alone as a distinguishing factor between HLH and sepsis, emphasizing the need to consider HLH in patients with persistent fever and hepatosplenomegaly.

Regarding laboratory parameters, aside from the diagnostic criteria for HLH, we observed that children with HLH had significantly higher levels of aspartate aminotransferase (117 vs. 41 U/L, *P* < 0.0001), lactate dehydrogenase (1113 vs. 555 U/L, *P* = 0.0002), and albumin (33.85 vs. 29.4 g/L, *P* = 0.0136) than those without HLH. Elevated liver enzyme levels, including aspartate aminotransferase, often manifest early in HLH. Although elevation of alanine aminotransferase levels is sensitive (present in 85% of patients with HLH), it lacks specificity^20,21^. Our data suggest that elevated aspartate aminotransferase and lactate dehydrogenase levels and low albumin levels when combined with other factors such as blood cell counts and triglyceride and ferritin levels can assist clinicians in evaluating HLH in children with sepsis.

Macrophage activation in HLH leads to increased fibrinolysis, resulting in elevated D-dimer levels, decreased fibrinogen levels, and prolonged coagulation panel results^20^. The present study findings demonstrated that hypofibrinogenemia was an independent predictor of HLH in children with sepsis. The clinical utility of fibrinogen level was further supported by our finding that a cutoff value of 2.43 g/L provided good sensitivity (72.41%) and specificity (76.27%) for identifying HLH in children with sepsis. This suggests that fibrinogen levels below 2.43 g/L serve as an early marker of HLH in pediatric sepsis cases. Notably, the HScore, which aids in identifying reactive HLH, includes a fibrinogen cutoff value of 2.5 g/L^21^. This consistency underscores the potential of hypofibrinogenemia as a distinguishing factor between HLH and sepsis, despite potential overlap with disseminated intravascular coagulation^22–27^. Prospective, multicenter research on fibrinogen cutoff values warrants further exploration.

Nonetheless, our study has certain limitations. The primary limitations of our study include its retrospective design and the absence of certain variables, such as bone marrow examination, natural killer cell function assays, and cytokine profiles. Wider-scale investigations are necessary to validate the clinical utility of fibrinogen in diagnosing HLH. Nonetheless, our findings indicate that decreased fibrinogen levels represent an independent predictor of HLH in pediatric sepsis. This suggests that reduced fibrinogen serves as an early diagnostic clue for HLH in pediatric sepsis cases.

## Methods

### Patient enrollment

Between January 2019 and June 2022, we retrospectively analyzed the medical records of pediatric patients with sepsis at Guangdong Women and Children Hospital. The clinical features and laboratory data at admission were extracted from the hospital information system, including both pediatric wards and intensive care units. The exclusion criteria comprised underlying medical conditions such as congenital heart disease, primary nephrotic syndrome, metabolic disorders, malignancies, immunodeficiency, or known autoimmune disorders. This study was approved by the Ethical Committee of Guangdong Women and Children Hospital, and all methods adhered to relevant guidelines and regulations (Ratification number: 202201283). Informed consent was obtained from legal guardians.

### Diagnostic criteria for sepsis and HLH

Sepsis was diagnosed based on The Third International Consensus Definitions for Sepsis and Septic Shock (Sepsis-3)^28^. HLH diagnoses adhered to the guidelines of the HLH-2004 Protocol of the Histiocyte Society^22^. Specifically, patients met at least five of the following eight criteria: fever, splenomegaly, cytopenia in ≥ 2 cell lineages, hypertriglyceridemia (≥ 265 mg/dL) and/or hypofibrinogenemia (< 150 mg/dL), hyperferritinemia (≥ 500 ng/mL), soluble CD25 level (> 2400U/mL), low or absent NK-cell cytotoxicity, and hemophagocytosis in bone marrow, spleen, or lymph nodes.

### Statistical analysis

We conducted all statistical analyses using SPSS 21.0 (SPSS Inc., Chicago, IL, USA). Unordered categorical variables were presented as numbers and percentages. Continuous variables were expressed as mean ± SD for normally distributed data or as median (quartiles) for skewed data. The Mann–Whitney U Test compared skewed continuous variables, and chi-square and Fisher’s exact tests analyzed unordered categorical variables. Multivariate binary logistic regression analysis investigated predictors of HLH in children with sepsis. Receiver operator characteristic curve analysis assessed the predictive value of HLH markers. Statistical significance was defined as *P* < 0.05.

### Ethics approval and consent to participate

This study was approved by the Ethical Committee of Guangdong Women and Children Hospital; all procedures were carried out in accordance with the relevant guidelines and regulations. Ratification Number: 202201283.

## Data Availability

The datasets analyzed during the current study are available from the corresponding author upon reasonable request.
